# Evaluation and Comparison of Two Different Cleaning Procedures on Mechanical Properties for Recemented Restoration Using Two Conventional Glass Ionomer Cement Types: An In Vitro Study

**DOI:** 10.7759/cureus.32044

**Published:** 2022-11-30

**Authors:** Reny K Ninan, Tripty Rahangdale, Saurabh Shrivastava, Prabha S Newaskar, Nishi Mishra, Karvika Nayak

**Affiliations:** 1 Prosthodontics and Crown & Bridge, Mansarovar Dental College, Bhopal, IND; 2 Prosthodontics, Rural Dental College, Pravara Institute of Medical Sciences Deemed to be University, Loni, IND; 3 Oral Medicine & Radiology, Mansarovar Dental College, Bhopal, IND

**Keywords:** gc fuji 1, recemented, carbide bur, prophy jet, ivoclar vivadent, gic cement, shear bond strength

## Abstract

Background

In modern dentistry, fixed dental prostheses are often the preferred and the most complex treatment procedures. However, the success of these procedures depends to a great extent on the durability and consistency of the ongoing reconstruction. This paper aimed to compare the shear bond characteristics of conventional glass ionomer cement types, namely, Vivaglass luting cement and GC fuji 1 cement, after being subjected to two different dentin-cleaning techniques: the tungsten carbide bur and the prophy jet. This would help determine whether the castings that have been cleaned and recemented are just as tenacious as when they were initially placed.

Methodology

A total of 60 human teeth extracted due to periodontal disease were collected for this study. The teeth were cleaned and stored in a thymol arrangement after carefully removing any leftover fragile tissue. They were randomly grouped as follows: luted with Vivaglass cement (N = 30) in group A and GC fuji 1 cement (N = 30) in group B. Each group's debonded surfaces were arbitrarily split into three subgroups, with each containing 10 samples. This was done to comply with dentin-cleaning techniques for recementation.

Results

The intergroup bond strengths calculated were compared among the subgroups. The mean strength for subgroup IA was 2.78 ± 0.24 MPa, subgroup IB was 2.60 ± 0.30 MPa, subgroup IIA was 4.39 ± 0.19 MPa, subgroup IIB was 3.80 ± 0.23 MPa, subgroup III A was 4.52 ± 0.22 MPa, and subgroup IIIB was 3.63 ± 0.17 MPa. An analysis of variance test showed there was a significant difference between the subgroups (F = 117.60; P = 0.001). The results revealed that there was a significant difference between the subgroups. Shear bond strength testing was performed using common testing equipment. The results indicated that there was not any significant difference between the mean increases in the shear bond strength of the two luting cement types. However, the GC fuji 1 cement showed greater initial shear bond strength than the Vivaglass cement. Additionally, when the tungsten carbide bur was used as a cleaning technique before recementing, it showed greater shear bond strength compared to the prophy jet.

Conclusions

The initial shear bond strength of GC fuji 1 (Group B) luting cement was slightly higher than that of Vivaglass (Group A), and the bond strength achieved after recementation with GC fuji 1 and surface cleaning with the carbide bur was the highest among the other three groups.

## Introduction

In modern dentistry, fixed dental prostheses is extensively used. However, these procedures are only effective as long as the restorations sustain and remain stable over time. The length and surface area of the crown, as well as the design of the tooth and the marginal seal, play important roles in achieving this [1,2]. To ensure that these restorations last, the correct luting cement (the substance used to bridge the gap between the tooth and the restoration) must be used [1,3]. Most of the time, the luting agent does not work well enough to keep the cement casting in place [4].

Biocompatibility, resistance to breakdown in oral fluids, good minor seal, thin layer thickness, low consistency, basic control, sufficient working time, and rapid setting are only some of the many advantages of using luting cement. High elasticity, maximum compressive and retentive strength, and strong adhesion to tooth or rebuild are only a few of the parameters that contribute to the luting cement's overall performance [5,6]. Glass ionomer cement is commonly used because it can synthetically bond to dentin and finish, release fluoride, and have a thermal expansion coefficient close to that of natural teeth [7]. To ensure their continued effectiveness and safety over the long term, authoritative repairs are highly dependent on the attachment [8]. When cement deposits are removed from the dentin, the connection between the dentin and glue framework strengthens as a result of improved adhesiveness. Revolving tools with pumice or puffs are commonly used to clear the smear layer and concrete accumulation, and they may effectively get rid of either the smear layer or the cement remnant [9]. The type of cement used and the way it was cleaned determined the tensile strength of permanent restorations [3].

This research aimed to compare the shear bond strengths of two commonly used glass ionomer cement, Vivaglass luting cement and GC fuji 1, after they have been subjected to two distinct dentin-cleaning procedures, namely, tungsten carbide bur and prophy jet, for the recementation of prostheses. Castings that were cleaned and recemented were tested to determine whether they retained their original strength.

## Materials and methods

A total of 60 teeth were extracted for this investigation because of periodontal disease. After the remaining soft tissue was physically removed, the teeth were cleaned and preserved in thymol solution until they were used. The molar tooth surface was facing upward when the teeth were mounted on self-curing acrylic resin (Pyrex quick repair denture base material, Roorkee, Uttarakhand, India; pink color; lot number 059/122) blocks. A piece of 12.5 × 30 mm polyvinyl chloride (PVC) pipe was designed for this purpose. The monomer and polymer components of an auto-polymerizing acrylic resin were combined in a porcelain jar at a volumetric ratio of 3:1 to create a resin that could be poured. The PVC pipe was used as a mold, and the teeth were inserted in the center of the mold until their roots were submerged in the acrylic resin and they were standing upright. Using a diamond bur (Microdont, Sao Paulo, Brazil; lot number 0791/18), the enamel layer was removed from the occlusal surface of the tooth specimen to create a 5 × 5 × 2 mm cavity in the superficial dentin surface of the middle of the tooth. Then, the same area was waxed with a blue inlay wax stick (Pyrex, Roorkee, Uttarakhand, India; lot number IW-004). After affixing 5 mm sprues to wax blocks and investing them in H&W investment material (Pyrex, Roorkee, Uttarakhand, India), the muffle was put inside a burnout furnace (the muffel furnace, India) and warmed to 800°C. Next, after a holding period of 40 minutes, the furnace was heated to 900°C, following which the investment material was cleaned off the metal blocks by blasting them with sand under a pressure of 6 bars. A universal silicone polishing kit was used to complete the metal blocks.

Grouping of teeth

The teeth were arbitrarily grouped as follows: Vivaglass cement luting (N = 30) and fuji 1 cement luting (N = 30).

The restoration was placed over a defined dentinal area and fitted with finger pressure. The luting concretes were applied to the base following the manufacturer’s guidelines before being set. The trailblazing tip was used to chisel away the set cement. After 24 hours in a 37°C thymol solution, the cemented samples were put through 1,000 heat cycles between 5°C and 55°C, with a 20-second rest period at each temperature. After being subjected to thermocycling again, the instances were reconstituted in the thymol configuration. Using standard testing equipment, the major shear bond strengths were measured after 12 hours. Application of a hub load using an etch-molded pole at a crosshead speed of 0.5 mm/minutes along the projecting concrete contact on a widely used testing machine could not establish a permanent shear bond. In each case, the highest load (in MPa) that caused the bond to break was also noted.

The debonded specimens of each group were randomly divided into three subgroups (10 specimens in each) according to the dentin-cleaning procedure used for recementation. Subgroup IA contained specimens that were resinified with Vivaglass cement and excavated; subgroup IB contained specimens reconsolidated with fuji 1 cement and cleaned with an excavator; subgroup IIA contained specimens resinified with Vivaglass cement and cleaned with tungsten carbide burs; subgroup IIB contained specimens resinified with fuji 1 cement and cleaned with tungsten carbide burs; subgroup IIIA consisted of specimens reconsolidated with Vivaglass cement and cleaned with prophy jet; and subgroup IIIB consisted of specimens reconsolidated with fuji type 1 cement and cleaned with prophy jet.

## Results

Intergroup bond strengths calculated for all subgroups were compared. The mean for subgroup IA was 2.78 ± 0.24 MPa, subgroup IB was 2.60 ± 0.30 MPa, subgroup IIA was 4.39 ± 0.19 MPa, subgroup IIB was 3.80 ± 0.23 MPa, subgroup IIIA was 4.52 ± 0.22 MPa, and subgroup IIIB was 3.63 ± 0.17 MPa. The ANOVA test result showed there was a significant difference between the subgroups with an F-value of 117.60 and a P-value of 0.001 (Table 1).

**Table 1:** Intergroup comparison of shear bond strength (MPa).

Group	Number of specimens	Mean (MPa)	Standard deviation	F-value	P-value
Vivaglass with excavator (IA)	10	2.78	0.24	117.60	0.001
Fuji 1 with excavator (IA)	10	2.60	0.30		
Vivaglass with tungsten carbide bur (IIA)	10	4.39	0.19		
Fuji 1 with tungsten carbide bur (IIB)	10	3.80	0.23		
Vivaglass with prophy jet (IIIA)	10	4.52	0.22		
Fuji 1 with prophy jet (IIIB)	10	3.63	0.17		
Total	60	3.62	0.76		

## Discussion

A luting chemical is used in a variety of dental procedures to attach dental prostheses and instruments to a patient’s teeth. Types of restorations include metal, ceramic, composite, or acrylic front teeth, supports, and pins. Some examples of luting cement include zinc phosphate, zinc oxide eugenol, zinc carboxylate, glass ionomer, pitch-altered glass ionomer, compomer, and tar cement. These types of cement are different from one another. In recent years, there has been a rise in the popularity of highly long-lasting dental prostheses. Nonetheless, the success of these methods heavily depends on the preservation and long-term safety of the rebuilding efforts. The length and surface area of the crown, the breadth of the crown at its narrowest point, the type of the tooth, and the minimal seal also affect the quality of restoration [1,2]. For high-quality restorations, luting cement must be used to fill the space between the tooth and the restoration, which makes the restoration last longer and stay in place [1,3].

If the luting cement doesn’t perform well, the cementation may fail. Microleakage inside the cement layer, spit or discharge during the cementation procedure, and hyperocclusion of the solidified prosthesis are all potential causes of cementation failure. Positive features of luting cement materials include biocompatibility, low dissolvability in oral liquids, a suitable negligible seal, a negligible film thickness, low consistency, basic control, and sufficient working time with the rapid set. The look of luting cement is also affected by how stiff it is, how strong it is in both compression and retention, and how well it sticks to the teeth or the restoration.

Depending on the trained professional’s material handling, the pre-treatment of the tooth surface, and the many-sided reconditioning, there may be several approaches to achieving this grip. Rigorous research has been conducted to ascertain the ability of cementing materials to determine the shear bond strength of fixed prosthodontics-supporting materials [1,8,9]. Repair cement’s shear bond strength can be affected by the cleaning process, which has been investigated in the literature [10-12]. This study aimed to compare the shear bond strength of two commonly used luting cement, Vivaglass and fuji 1 cement, before performing any successful reconstruction operation. Subsequently, tungsten carbide bur or prophy jet was used to clean the area. Finally, the shear bond strength of the recemented restoration was tested. Liquid copolymers of weak polyalkenoic acids such as itaconic, maleic, and carboxyl react with aluminum fluorosilicate glass particles to fix the cement material. Adding tartaric acid to the powder helps increase the flow rate and the powder’s shelf life [13]. These acids can also be added to the powdered component as freeze-dried forms and reconstituted with water. Enamel and dentin apatite include calcium and phosphate, and some experts believe that carboxyl groups in the destructive dilemma pose a threat to these particles. The concentration is mostly on improving compressibility (90-230 MPa). In 1997, Bandgar et al. [14] utilized cold fix tar to encase the teeth in an acrylic block, rendering the apparatus portable and easy to include in a larger shear bond strength testing rig. Paul et al. [33] subjected dentin to heated cycling and maintained a constant intra-pulpal tension.

This study concluded that a fixed size of 5 × 5 × 2 mm was the most practical option. Aspects of 4 × 4 × 2 mm were selected for occurrences to mimic the above lab-treated durable recoveries, as was done in previous research [15,16]. In vitro testing of the luting cement maintenance should take into account the fact that oral stickiness has been shown to significantly alter the adhesive and mechanical qualities of luting cement. Before submitting models for flexible testing according to ISO standard 10477 [17], the two luting cement types were allowed time to cure in wet circumstances. Pazinatto et al. [18] conducted their study at a normalized temperature range of 5-55°C for 15 seconds. These limits appeared to be safe for usage in clinical settings, and oral tissues were found to tolerate them well. Osvaldo et al. [19] immersed their research samples in water for 30 seconds at a time at temperatures ranging from 5°C to 55°C, with a total of 200-37,500 warm cycles. For the previous analysis, instances were subjected to 1,000 intensity cycles at 5-55 °C with a 20-second stay length. To measure shear bond strength, this study utilized industry-standard testing equipment. Some studies have suggested that constraining characteristics exceed 25 MPa, which is typical of fewer cement frameworks, but regular tests, such as the shear bond strength test, have limitations. Pressure concentrated at the holding surface may break the link prematurely, leading to deformation, as suggested by Powers et al. [20]. Piwowarcryk et al. [8] found that fuji 1 cement restored the bond quality of instances ranging from 4.56 to 5.26 for shear holding. It is important to keep in mind that different materials and testing methods may make drawing firm conclusions from the written word difficult. The results showed that the benefits of shear bond strength for the two luting experts were completely different. Although the recementing process may seem like the main stage of cementation, any surplus cement compromises the credibility of the group. When surplus concrete is removed, bond strength increases [21,22]. The most common method of cleaning involves cleaning instruments with a prophy jet, and their effect on the smear layer and temporary cement remnants ranges from partial to total eradication of waste or smear layer. Koodaryan et al. [10] should be cited as a relevant work.

Extra cement was removed using a tungsten carbide bur, and the resulting Vivaglass cement repairs showed just a marginal drop in bond quality compared to the original shear bond strength. Additionally, shear bond strength was reduced when surplus cement was removed with a prophy jet and then recemented with Vivaglass cement. When a tungsten carbide bur was used to remove excess cement from Vivaglass cement samples, the shear bond strength was significantly stronger than when a prophy jet was used. Excess cement was removed with a tungsten carbide bur and then recemented with fuji 1 cement, resulting in a significantly lower bond quality compared to the initial shear bond strength. These samples also show that the bond quality of glass ionomer cement that was recoated following prophy jet evacuation of excess cement was lower compared to the initial shear bond strength. When comparing the shear bond strength of prophy stream-recemented and tungsten carbide burs-recemented fuji 1 cement, the latter led to a stronger shear bond strength. The results showed that the benefits of shear bond strength documented for the group in which surplus cement was removed using a tungsten carbide bur and recoated with fuji 1 cement were much greater than those obtained for other groups.

Initial shear bond strength testing showed that fuji 1 cement is superior to Vivaglass cement. When tungsten carbide burs are used as a pre-recement cleaning procedure, the shear bond strength is significantly higher compared to the use of a prophy jet for the cleaning procedure. The implications of this cement accumulation on bond strength over shorter time periods require further investigation.

## Conclusions

This study found that the initial shear bond strength of the fuji 1 luting cement (Group B) was slightly higher than that of Vivaglass (Group A), that the bond strength of both groups was higher when the surfaces were prepared with a tungsten carbide bur rather than a prophy jet, and that the highest shear bond strength was achieved after recementation using fuji 1 cement.
